# Childhood maltreatment and vulnerability to substance use disorders: The mediating role of psychological security

**DOI:** 10.34172/hpp.42525

**Published:** 2024-07-29

**Authors:** Behzad Shalchi, Maryam Nosrati Beigzadeh, Ali Reza Shafiee-Kandjani, Hassan Shahrokhi, Behnaz Hoseinzadeh Khanmiri

**Affiliations:** ^1^Research Center of Psychiatry and Behavioral Sciences, Tabriz University of Medical Sciences, Tabriz, Iran; ^2^Working Group of Psychiatry and Psychology Culture-based Knowledge Development, Tabriz University of Medical Sciences, Tabriz, Iran

**Keywords:** Child maltreatment, Psychological security, Substance use disorders

## Abstract

**Background:**

Understanding the factors associated with the propensity for drug dependence might be helpful in providing the best strategies for substance use prevention among youth. The present study was conducted to examine the association between childhood maltreatment (CM) and susceptibility to substance use disorders (SUDs), taking into account the mediating role of psychological safety.

**Methods:**

In this cross-sectional study, multistage cluster sampling was employed to recruit 400 male students from the three universities of Tabriz in 2019. Research instruments included the valid and reliable scales of substance abuse, childhood trauma, and mental safety. Data were analyzed using Pearson correlation, and structural equation modeling.

**Results:**

The results showed positive significant relationships between vulnerability to SUDs, CM, and low levels of psychological safety. The evaluation of our hypothetical research model using fit indices showed that the model fits well the measurement model (CFI=0.97, GFI=0.95, RMSEA=0.058).

**Conclusion:**

Our proposed theoretical model suggested psychological security as a mediator between CM and SUDs. CM explained addiction in college students through reducing psychological security. Investigating these interactive processes seems to be detrimental, considering that they may enhance our understanding of the ways to reduce the risk.

## Introduction

 Childhood maltreatment (CM) experiences might have negative effects on various aspects of development, including mental health, physical health, and social functioning.^1^ CM is considered as a global problem that may lead to all sorts of unfortunate consequences. Unpleasant childhood experiences are associated with many physical and mental illnesses, abnormalities, and unhealthy lifestyles in adulthood. The role of CM experience in creating cognitive vulnerabilities has been clarified in various statistical models.^2,3^ CM includes all types of abuse and neglect that may endanger the child’s health and development.^4^ Experiencing any traumatic event, including emotional abuse, physical abuse, and sexual abuse in childhood can affect people’s well-being.^5^

 It appears that the combination of adverse childhood experiences, neglect, patterns of maltreatment (abuse), and family dysfunction (parental substance abuse, incarceration, separation, mental illness, and domestic violence) during childhood might lead to risky behaviors and negative outcomes in subsequent life courses.^6^ Adverse life experiences are linked to psychological trauma that may last a lifetime.^7^ Researches have indicated that a greater frequency of adverse childhood experiences is substantially linked to a larger probability of unfavorable consequences during adolescence and adulthood.^8,9^ Adverse childhood experiences have been linked to unfavorable consequences, such as substance misuse and social, emotional, and cognitive deficits, as well as the adoption of dangerous health behaviors.^10-12^

 One of the domains that is particularly affected by CM is the vulnerability to substance use disorder (SUD), which is a chronic and relapsing condition characterized by compulsive drug seeking and use, despite harmful consequences.^13^ Previous studies have shown that the individuals who experience maltreatment in childhood are more likely to develop SUD in adolescence or adulthood, compared to those who do not have such experiences.^14^ However, the mechanisms that explain how CM leads to SUD are not fully understood. According to neuroscience research, stress may alter the way that brain works and looks, which may lead to drug-seeking behavior.^15^ Drug misuse in adulthood has been shown to be linked to the experience of unfavorable conditions throughout childhood and adolescence.^6,16,17^ The most significant mediators of the tendency to participate in dangerous behaviors are the quality of the environment and the ability of parents or caregivers to raise their children.^18^ According to a study on over 10 000 high-risk adolescents, the most significant protective and enhancing elements in substance addiction prevention programs were parent-child connection, parental supervision versus punishment, and family interactions.^19^ These three family characteristics have the highest protective effects against a number of negative outcomes, including delinquency, substance addiction, academic failure, and adolescent pregnancy, even though the association with peers who use drugs was reported to be the most predictive risk factor for substance misuse.^20^ These protective mechanisms can be promoted by parents or successful family-centered treatments.^21^

 According to research, children are more likely to have behavioral problems if they have more risk factors or family issues. Poor parenting and socialization practices,^22^ ineffective supervision,^23^ ineffective discipline practices, high levels of negativity/reinforcement, parenting styles, conflict over parenting techniques, harsh corporal punishment, low parental attachment, lack of cooperation and companionship, and a negative family environment are all examples of family factors associated with substance abuse.^24-26^ CM can have profound and lasting effects on a person’s psychological security in adulthood.^2,3^ The trauma experienced in childhood, either physical, emotional or sexual abuse, can negatively shape a person’s self-esteem, trust in others and general sense of safety and security.^5,27^ Psychological security is considered to be a basic human need that is essential for optimal development and well-being.^28^

 Individuals who feel psychologically secure usually perceive that the world is emotionally secure or free from emotional harm.^29^ They usually have high confidence and trust in themselves and others, feel less anxious, and tend to be more social and actively involved in relationships with others.^29^ People who feel psychologically secure may not perceive the world and other people as a threat. They also do not feel the threat that they might easily be hurt by other people’s emotional behaviors; thus, they strive to undertake difficult tasks, and also take risk to attain higher goals in life.^30^ However, psychological security can be undermined by exposure to maltreatment, especially when it occurs within the family context. Research has shown that less family emotional support is one of the main factors that may decrease psychological security.^31^

 As a necessary component of existence, psychological safety provides equilibrium and stability, and serves as a buffer against hopelessness and dangerous surroundings.^32^ It appears when a youngster perceives the outside world as a dependable and secure environment.^33^ Numerous studies have demonstrated the preventive role of psychological safety against various forms of childhood adversity, such as child sexual abuse,^34^ parental sadness,^35^ and challenging behavior.^36^ Maltreated children may develop insecure attachment styles, low self-esteem, poor emotion regulation, and negative cognitive schemas, which can all impair their ability to form and maintain healthy relationships later in their life. These deficits in psychological security may increase the risk of SUD, as individuals may seek to cope with their emotional distress and unmet needs through substance use.^16^

 The aim of this study was to examine the mediating role of psychological security in the relationship between CM and SUD. We were going to propose a theoretical model that may add evidence to the literature on the effects of maltreatment on psychological security and the effects of psychological security on SUD. We also reviewed the empirical evidence that supports the model and suggest directions for future research and implications for prevention and intervention. Enhancing psychological security may be a key strategy to reduce the vulnerability to SUD among individuals who have experienced CM. While psychological and environmental factors are significant predictors of substance misuse inclinations, little is known about how CM interacts with these elements to increase susceptibility. This study may be helpful in increasing our knowledge of the preventive factors by offering a new perspective on the connection between vulnerability to drug misuse and maltreatment among youth.

## Materials and Methods

 In this cross-sectional study, the statistical population were all male students of the three universities of Tabriz, Shahid Madani Azarbaijan and Islamic Azad University of Tabriz who were studying in the academic year of 2019. Based on the formula of Tabachnick and Fidell, the following formula was used to calculate the sample size (N = 400), depending on the number of predictor variables: N > 50 + 8M (number of predictor variables = M). Multistage cluster sampling was used to recruit 400 students in Tabriz universities. One faculty was selected from each 3 universities, and at the next stage, one educational group was considered from each faculty. Finally, the students of five classes from the selected educational groups were invited to participate in the study.

###  Participants and procedure

 The criteria for participation in the study were male students aged 18 to 23 years (young adults). The specific cultural conditions in the study area influenced the selection of the sample, as Iranian girls in this geographical area (Tabriz city) are influenced by the traditional family structure and are therefore less likely to report experiences of abuse.

 The following instruments were used in the present study:

###  Childhood Trauma Questionnaire (CTQ)

 This questionnaire was developed by Bernstein et al^37^ who provided the final 53-item version.^38^ It measures maltreatment on five subscales and yields a total score indicating global maltreatment. These five scales are emotional abuse, physical abuse, sexual abuse, emotional neglect, and physical neglect. Items are rated on a 5-point Likert scale (1 = strongly disagree to 5 = strongly agree). Bernstein et al approved the reliability of the questionnaire by two methods of retesting and Cronbach’s alpha ranging from 0.79 to 0.94.^37^ In Iran, Mikaeili and Zamanloo used the factor analysis method to confirm the validity of this questionnaire. The factors obtained were consistent with the subscales of the questionnaire.^39^ In the present study, the total calculated Cronbach’s alpha value was 0.80.

###  Psychological Security Questionnaire 

 This questionnaire was developed by Maslow et al^40^ and standardized by Aminpour.^41^ The abbreviated student form has 18 items in 4 domains: self-confidence, feeling of dissatisfaction, incompatibility with the environment, and people’s view of themselves. This questionnaire can be used to identify and measure the personal characteristics that create feelings of security and insecurity, and to identify individuals who are psychologically and emotionally insecure. In the present study, the calculated Cronbach’s alpha values for self-confidence, dissatisfaction, incompatibility with the environment, and people’s view of themselves were 0.77, 0.75, 0.80, and 0.79, respectively.

###  Substance Abuse Questionnaire

 This questionnaire was provided by Anisi et al in Tehran.^42^ The questionnaire includes 75 items and 4 factors: depression and helplessness, positive attitude toward substances, anxiety and fear of others, and sensation seeking. Scoring is based on a 4-point Likert scale (strongly disagree, disagree, partially agree, and agree). The total score of the questionnaire is the sum of all items scores. The score range of the questionnaire ranges from 0 to 225. The cut-off value for the total score of the questionnaire is 80. In other words, persons with a score of 80 or more are at risk of addiction. The reliability of the test was calculated as 0.97 according to Cronbach’s alpha. To confirm its validity, the correlation of the test with the Zuckerman’s Depression (0.76), Anxiety (0.71), Stress (DASS) (0.77), and Sensation Seeking (0.78) scales was examined.^42^ An alpha of 0.83 was calculated for this questionnaire in the present study.

###  Statistical analysis 

 Pearson correlation coefficient test and structural equation modeling were used for data analysis. Chi-squared, ratio of chi-squared to degree of freedom, goodness of fit, adaptive goodness of fit, comparative fit, mean square error of approximation, and mean square of residual were used to examine model fit. If the chi-square is not statistically significant, it means that the fit is very good. However, since this value is often obtained from samples with more than 100 significant values, it is not appropriate to measure the fit of the model. If the ratio of chi-squared to degree of freedom is less than 3, this indicates a very good fit. When comparative fit index (CFI), adjusted goodness of fit index (AGFI), and goodness of fit index (GFI) are greater than 0.90 and root mean square error of approximation (RMSEA) and root mean square residual (RMR) are less than 0.05, this is considered a very good and reasonable fit, and less than 0.08 is considered a good and reasonable fit Data were analyzed using SPSS version 26 and LISREL 10 software. Pearson correlation and structural equation modeling were used for data analysis.^43^

## Results

 The age range of the participants was from 18 to 32 years, with a mean of 21.96 years and a standard deviation of 2.18. About 46% of the subjects studied in the fields of technical and engineering, 20.8% studied basic sciences, 32.5% studied humanities, and 0.3% studied arts. Descriptive data on parents’ education level in the whole sample were as follows: illiterate (father [F]: 5.5%, mother [M]: 12.5%); elementary school (F: 11%, M:11.8%); high school (F: 16%, M:15.3%); diploma (F: 33.3%, M: 38.8%); associate degree (F: 6.8%, M: 5.5%); B.A. (F: 16%, M: 10.3%); M.A. (F: 8.3%, M: 2.5%); and Ph.D. (F: 3.3%, M: 3.3%). The economic status of the families of the study participants, who were divided into five groups, was as follows: very poor (8.3%); poor (4.8%); moderate (56.8%); good (22.5%); and very good (6%). Descriptive statistics for the variables studied are presented in Table 1.

 As shown in Table 2, “vulnerability to SUDs” and its components are positively and significantly correlated with “CM” and its components. Among the components of “susceptibility to SUD”, only “high sensation seeking” was not significantly correlated with “CM” components.

 As shown in Table 3, “vulnerability to SUDs” and its components are negatively and significantly correlated with “psychological safety” and its components. Increasing “psychological safety” decreases the likelihood of “vulnerability to SUDs” and vice versa.

 As shown in Table 4, the components of “CM” are negatively and significantly correlated with “psychological security” and its components. Reducing “CM experiences” increases the possibility of “psychological security” and vice versa.

**Figure 1:**
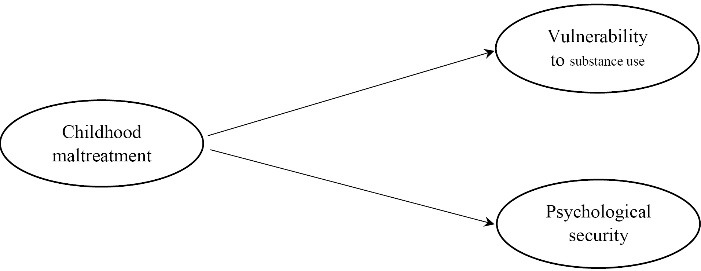
The hypothetical conceptual model in the present study

**Figure 2:**
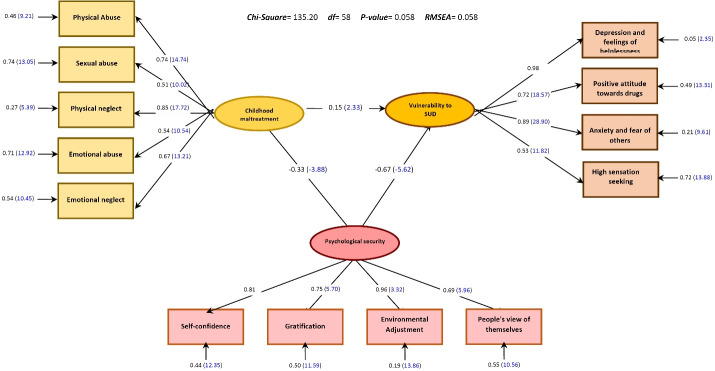
Standardized and (t) values of the finalized structural model (N = 400)

 As shown in Table 5 and Figure 1, model fit indices showed that the model fits well the measurement model (CFI = 0.97, GFI = 0.95, RMSEA = 0.058). The finalized structural model is also presented in Figure 2.

**Table 1:** Mean and standard deviation of studied variables

**Variables**	**Mean**	**Standard deviation**
Depression and feelings of helplessness	36.25	16.23
Positive attitude towards drugs	17.98	12.31
Anxiety and fear of others	22.41	9.28
High sensation seeking	17.04	5.51
Vulnerability to SUD	93.69	37.62
Physical abuse	3.58	3.94
Sexual abuse	3.85	4.20
Physical neglect	6.12	4.15
Emotional abuse	3.76	3.67
Emotional neglect	8.61	5.37
Childhood maltreatment	25.93	16.22
Self-confidence	5.26	1.90
Gratification	1.58	0.97
Environmental adjustment	3.31	1.27
People's view of themselves	1.91	0.89
Psychological security	12.07	3.35

**Table 2:** The correlation matrix of vulnerability to SUD and childhood maltreatment

**Variables**	**1**	**2**	**3**	**4**	**5**	**6**	**7**	**8**	**9**	**10**	**11**
1) Depression and feelings of helplessness	1										
2) Positive attitude towards drugs	0.69^**^	1									
3) Anxiety and fear of others	0.86^**^	0.62^**^	1								
4) High sensation seeking	0.50^**^	0.43^**^	0.50^**^	1							
5) Vulnerability to SUD	0.94^**^	0.84^**^	0.90^**^	0.63^**^	1						
6) Physical Abuse	0.26^**^	0.28^**^	0.25^**^	0.04	0.27^**^	1					
7) Sexual abuse	0.20^**^	0.25^**^	0.17^**^	0.01	0.21^**^	0.57^**^	1				
8) Physical neglect	0.24^**^	0.29^**^	0.18^**^	-0.03	0.24^**^	0.38^**^	0.32^**^	1			
9) Emotional abuse	0.28^**^	0.30^**^	0.28^**^	0.07	0.30^**^	0.62^**^	0.58^**^	0.62^**^	1		
10) Emotional neglect	0.32^**^	0.29^**^	0.27^**^	0.05	0.31^**^	0.37^**^	0.35^**^	0.37^**^	0.41^**^	1	
11) Childhood maltreatment	0.35^**^	0.37^**^	0.30^**^	0.03	0.35^**^	0.76^**^	0.73^**^	0.74^**^	0.79^**^	0.76^**^	1

**P<0.01, *P<0.05

**Table 3:** The correlation matrix of vulnerability to SUD and psychological security

**Variables**	**1**	**2**	**3**	**4**	**5**	**6**	**7**	**8**	**9**	**10**
1) Depression and feelings of helplessness	1									
2) Positive attitude towards drugs	0.69^**^	1								
3) Anxiety and fear of others	0.86^**^	0.62^**^	1							
4) High sensation seeking	0.50^**^	0.43^**^	0.50^**^	1						
5) Vulnerability to SUD	0.94^**^	0.84^**^	0.90^**^	0.63^**^	1					
6) Self-confidence	-0.35^**^	-0.16^**^	-0.29^**^	-0.10^*^	0.29^**^-	1				
7) Gratification	-0.34^**^	-0.21^**^	-0.29^**^	-0.15^**^	-0.31^**^	0.18^**^	1			
8) Environmental adjustment	-0.16^**^	-0.02	-0.14^**^	0.07	-0.10^**^	0.38^**^	0.09^*^	1		
9) People's view of themselves	-0.38^**^	-0.29^**^	-0.29^**^	-0.15^**^	-0.35^**^	0.21^**^	0.32^**^	0.07	1	
10) Psychological security	-0.46^**^	-0.24^**^	-0.38^**^	-0.11^**^	-0.39^**^	0.82^**^	0.51^**^	0.64^**^	0.51^**^	1

**P<0.01, *P<0.05

**Table 4:** The Correlation matrix of childhood maltreatment and psychological security

**Variables**	**1**	**2**	**3**	**4**	**5**	**6**	**7**	**8**	**9**	**10**	**11**
1) Physical Abuse	1										
2) Sexual abuse	0.58^**^	1									
3) Physical neglect	0.39^**^	0.33^**^	1								
4) Emotional abuse	0.63^**^	0.59^**^	0.47^**^	1							
5) Emotional neglect	0.38^**^	0.35^**^	0.62^**^	0.42^**^	1						
6) Childhood maltreatment	0.76^**^	0.73^**^	0.75^**^	0.79^**^	0.77^**^	1					
7) Self-confidence	-0.07	-0.03	-0.05	-0.13^*^	-0.21^**^	-0.14^**^	1				
8) Gratification	-0.07	-0.15^**^	0.01	-0.11^*^	-0.12^*^	-0.12^*^	0.18^**^	1			
9) Environmental adjustment	-0.06	-0.13^*^	-0.06	-0.10^*^	-0.11^*^	-0.12^*^	0.39^**^	0.10^*^	1		
10) People's view of themselves	-0.18^**^	-0.11^*^	-0.08	-0.19^**^	-0.18^**^	-0.20^**^	0.22^**^	0.32^**^	0.07	1	
11) Psychological security	-0.14^**^	-0.14^**^	-0.07	-0.19^**^	-0.24^**^	-0.21^**^	0.82^**^	0.52^**^	0.65^**^	0.51^**^	1

**P<0.01, *P<0.05

**Table 5:** Model fit indices presented in the research

**Index of fit**	**Indicator values**
Chi-square (χ^2^)	135.20
χ^2^/*df*	2.33
Goodness of Fit Index (GFI)	0.95
Adjusted Goodness of Fit Index (AGFI)	0.95
Comparative Fit Index (CFI)	0.97
Root Mean Square Error of Approximation (RMSEA)	0.058
Root Mean Square Residual (RMR)	2.32

## Discussion

 In the present study, we proposed and reviewed a theoretical model that suggests psychological security as a mediator between CM and SUD. According to this model, CM undermines the development of psychological security, which is a sense of safety, trust, and confidence among individuals. Psychological security, in turn, protects against the risk of SUD, as it enables individuals to cope with stress, regulate emotions, and seek support in healthy ways.^44^ We also discussed the empirical evidence that supports the model, as well as the implications for prevention and intervention. Our results were consistent with the theoretical underpinnings and conclusions of related research, and the hypothesized model developed in this paper fits the experimental data well.

 The present study was conducted on young students between the ages of 18 and 23. A population that was experiencing separation from family, and entry into a large university community for the first time, while being exposed to new academic, professional and emotional pressures. Studies show that the students who come to university with feelings of loneliness, dependent on their parents and lacking psychological security suffered from clinical problems (physical and psychological symptoms) and academic failure during their studies.^45^ On the other hand, previous studies have shown that people who have had negative experiences in their childhood are more likely to be more susceptible to new pressures and stress in young adulthood and are more likely to use drugs.^13,16,17,46^ According to the latest statistics in Iran, the mean age of drug use initiation among men is 24.1 years.^43^ Therefore, this study examined the tendency to use drugs before reaching this age. In terms of gender, 95% of addicts in Iran are men,^44^ which firmly suggests that Iranian boys are more prone to drug addiction, so the study of boys younger than 24 years of age were considered as the priority of our research.

 The remarkable correlation between the tendency to use drugs and CM in the present study can be interpreted in the light of research findings^47^ and the National Epidemiological Survey on Alcohol and Related Conditions (NESARC) epidemiologic study conducted in the United States, which indicates that CM increases the likelihood of alcohol abuse in adulthood.^48^ Previous research has shown that adolescents who are delinquent and have a history of drug dependence have experienced physical and sexual abuse, neglect, criminal victimization, violence, and other traumatic events.^49,50^ In this context, a previous study found a favorable correlation between drug dependence and sexual and domestic violence.^47^ In another study, emotional, sexual, and physical abuse were reported as the leading causes of SUD.Children and adolescents can often cope with one or two problems within their families; however, the likelihood of substance abuse increases when there are frequent family problems.^51,52^ According to another research, the strong parent-child bond helped Mexican-American teens to become more resilient and less likely to commit crimes.^53^ Adolescents and young adults who are exposed to a series of stressful events may have difficulties in controlling their emotions and thus feel confused, powerless, and unstable.^54^ Substance abuse can be a means of escaping or alleviating the distress, worry, and anger associated with such situations. However, it is impossible to ignore the damaging consequences of even the most minor childhood abuse. According to one study, “minor” forms of maltreatment can have a significant impact on the likelihood of substance misuse.^55^

 In contrast to previous studies, the current study found no significant association between CM and the variable of high need for arousal, a factor associated with the propensity to use drugs. In the view of this result, it can be said that arousal need might be an innate personality trait which is influenced by heredity. Therefore, bad experiences in the past have a low influence on mood characteristics, such as arousal need.^56^ Furthermore, our results showed that CM has a negative relationship with psychological security. In this way, the children who are exposed to more maltreatment feel less psychologically safe and secure. This finding is consistent with those found in Al-Anani studiy^57^ and the studies that showed psychological security as a protective factor in exposure to adverse childhood experiences.^32,33^ Individuals whose basic needs, including physical needs, are neglected seem to be unable to fulfil higher needs, such as safety. Also, the individuals who are frequently neglected and harassed may experience high levels of anxiety and stress, and may perceive the world as an unsafe place. The persistence of such conditions causes the individuals to experience negative emotions that they may not be coped with, so they might not trust others, and thus withdraw from social activities.

 Although we found no study on the relationship between psychological safety and the tendency to abuse drugs, the results of some studies have shown that unhealthy and tense atmosphere in the families may significantly threaten, and even sometimes completely disrupt psychological security of the child.^54,58^ Another study has shown that the mothers with unresolved intrapersonal and interpersonal problems might disrupt their child’s psychological security and cause insecure attachment, which may consequently put the child at risk of displaying unhealthy behaviors, like impulsive behaviors, in adolescence.^59^ Several previous studies have also shown that some factors related to psychological security, such as secure or insecure attachments,^60^ attachment styles to parents,^61^ and self-esteem,^62^ can predict drug use or nonuse in adolescents and youth. In other words, the individuals with secure attachments and appropriate interactions with others, particularly their parents, might cope more easily with daily problems, compared to their counterparts. In contrast, the individuals with insecure attachment styles may consider substance abuse as an emotional alternative, because they feel insecure and lonely. All such findings support the mediating role of psychological security that was identified proposed in the present study. According to our model, psychological security has a mediating role in reducing the impact of CM, and seems to control the tendency to abuse drugs in individuals exposed to such negative experiences. Consistent with this finding, a previous study showed that some individuals respond flexibly to such experiences, and achieve adaptive levels of performance, despite the great psychological risks caused by CM.^63^ In this context, other studies have shown that adaptive psychological performance,^62^ cognitive factors,^64^ and social and supportive factors are potential protective factors against the consequences of CM, resulting in a lower risk of substance abuse propensity.^65^

 According to this model psychological security variables that have been suggested as acceptable mediators include self-confidence, satisfaction, adjustment to environment, and others’ views, each of which had a significant reducing effect size in the correlation matrix of CM and SUD in the present study. This indicates that CM survivors in this study who reported higher psychological security were less likely to use drugs. Previous studies whose findings were consistent with those of our study have shown that traits such as perseverance,^66^ self-confidence,^67^ and having positive attitude towards the future^68^ may lead to better health among individuals with CM experiences.

 Our model is consistent with the developmental psychopathology framework, which emphasizes the complex and dynamic interactions between risk and protective factors across the lifespan.^69^ The model also provides us with evidence on the effects of maltreatment on attachment, self-esteem, emotion regulation, and cognitive schemas, which are key components of psychological security.^70^ Our model also supports the self-medication hypothesis, which posits that individuals might use substances to alleviate their psychological distress and unmet needs.^71^ The model has several strengths and limitations. As one strength, it provides a comprehensive and parsimonious explanation for the link between CM and SUD. It is also supported by empirical evidence from various sources, such as cross-sectional, longitudinal, and experimental studies.^62-71^ Our model has important implications for prevention and intervention of SUD among individuals who have experienced CM. Prevention programs should aim to reduce the occurrence and impact of maltreatment, as well as to promote the development of psychological security among children and adolescents. Intervention programs should focus on enhancing psychological security among adults who suffer from SUD, by providing them with a safe and supportive medium.

 Although CM was found to be a strong predictor of SUD,^14^ the results of our study provided insight into the protective role of psychological security. While “prevention” is primarily viewed as an attempt to reduce risk, improving psychological safety through enhancing self-confidence and environmental adjustment seems to be one of the most effective preventive interventions for risky behaviors among adolescents.

## Limitations

 One limitation of our study was the use of self-report instrument. Many individuals tend not to answer questions about their experiences of abuse, such as sexual abuse. Therefore, the sections related to sexual abuse must be omitted from many studies. In this context, it is suggested to use other data collection methods, such as interviews, to obtain useful data. The limited sample size (male students) in this study makes it impossible to generalize the results to female populations. The particular cultural conditions in the studied area influenced our sample selection, as Iranian girls in this geographical area (the city of Tabriz) are influenced by the traditional family structure, and have therefore low willingness to report their experiences of abuse. Meanwhile, the model is mostly based on correlational data, which limits the causal inference and the direction of the effects. Another limitation is that the model does not account for the heterogeneity and diversity of maltreatment and SUD phenomena, such as the types, severity, and timing of maltreatment, as well as the substances, and their use patterns and consequences. Future research should address these limitations and test the model using more rigorous methods, such as randomized controlled trials, prospective longitudinal designs, or multilevel analyses. Future research should also examine the potential moderators and mediators of the model, such as genetic factors, personality traits, social support, and coping skills. Moreover, the applicability and generalizability of the model to different populations and contexts should be examined.

## Conclusion

 In the present study, we proposed a theoretical model that suggests psychological security as a mediator between CM and SUD. According to this model, CM undermines the development of psychological security, which is a sense of safety, trust, and confidence in individuals and others. Psychological security, in turn, protects against the risk of SUD, as it enables individuals to cope with stress, regulate emotions, and seek support in healthy ways. We also discussed the empirical evidence that supports the model, as well as the implications for prevention and intervention. The proposed model was identified in good agreement with the data. CM explained addiction in young boys through reducing psychological security. Investigating these interactive processes seems necessary as they enhance our understanding of the ways to reduce risk. Psychological security was identified to be an influential mediator in the relationship between CM and SUD in Iranian young boys. Investigating other protective and mediating factors to identify the long-term effects of CM in different cultures needs further research. Moreover, identifying the mediating factors that are less dependent on context, cognitive ability, and/or environmental characteristics is a promising avenue for future interventions among individuals at risk for SUD.

## Acknowledgements

 We appreciate All student for participating in this study.

## Ethical Approval

 All procedures performed in studies involving human participants were in accordance with the ethical standards of the institutional research committee and with the and its later amendments or comparable ethical standards. (Ethics code: IR. TBZMED. REC. 1395.1182) in Tabriz University of Medical Sciences.
